# KCa3.1 Inhibition Switches the Astrocyte Phenotype during Astrogliosis Associated with Ischemic Stroke Via Endoplasmic Reticulum Stress and MAPK Signaling Pathways

**DOI:** 10.3389/fncel.2017.00319

**Published:** 2017-10-12

**Authors:** Zhihua Yu, Mengni Yi, Tianjiao Wei, Xiaoling Gao, Hongzhuan Chen

**Affiliations:** Department of Pharmacology and Chemical Biology, Shanghai Jiao Tong University School of Medicine, Shanghai, China

**Keywords:** astrocytes, mouse, GFAP, ischemia, calcium

## Abstract

Ischemic stroke is a devastating neurological disease that can initiate a phenotype switch in astrocytes. Reactive astrogliosis is a significant pathological feature of ischemic stroke and is accompanied by changes in gene expression, hypertrophied processes and proliferation. The intermediate-conductance Ca^2+^-activated potassium channel KCa3.1 has been shown to contribute to astrogliosis-induced neuroinflammation in Alzheimer’s disease (AD). We here present evidence, from both astrocytes subjected to oxygen–glucose deprivation (OGD) and from the brains of mice subjected to permanent middle cerebral artery occlusion (pMCAO), that KCa3.1 represents a valid pharmacological target for modulation of astrocyte phenotype during astrogliosis caused by ischemic stroke. In the primary cultured astrocytes, OGD led to increased expression of KCa3.1, which was associated with upregulation of the astrogliosis marker, glial fibrillary acidic protein (GFAP). Pharmacological blockade or genetic deletion of KCa3.1 suppressed OGD-induced up-regulation of GFAP, endoplasmic reticulum (ER) stress marker 78 kDa glucose-regulated protein (GRP78) and phosphorylated eIF-2α through the c-Jun/JNK and ERK1/2 signaling pathways. We next investigated the effect of genetic deletion of KCa3.1 in the pMCAO mouse model. KCa3.1 deficiency also attenuated ER stress and astrogliosis through c-Jun/JNK and ERK1/2 signaling pathways following pMCAO in *KCa3.1^−/−^* mice. Our data suggest that blockade of KCa3.1 might represent a promising strategy for the treatment of ischemic stroke.

## Introduction

Ischemic stroke is a devastating neurological disease and is a major cause of death and severe disability. Currently, the only approved pharmacological therapy for ischemic stroke is recombinant tissue plasminogen activator (tPA; Alberts and Naidech, 2013) but the effect of tPA is limited by a narrow therapeutic window (<4.5 h; Fang et al., 2010).

All types of brain cell, including glial cells, vascular cells and neurons, are affected during ischemic stroke but, over the past two decades, most clinical trials in stroke patients have focused only on neurons. New therapeutic approaches benefiting multiple cell types must be considered. As mediators of neurological recovery and neurovascular plasticity, astrocytes may contribute to neural repair and the stabilization of synapses during synaptogenesis (Sofroniew, 2005). Ischemic stroke can initiate a phenotypic switch in astrocytes and, during reactive astrogliosis, the cells exhibit hypertrophied processes and produce proinflammatory cytokines.

KCa3.1, the intermediate-conductance Ca^2+^-activated K^+^ channel, is involved in the activation of many cell types, including immune cells, smooth muscle cells and fibroblasts (Bi et al., 2013; Chiang et al., 2017; Oliván-Viguera et al., 2017; Staal et al., 2017). Activation of KCa3.1 regulates potassium efflux, membrane hyperpolarization and calcium influx during cellular activation (Ghanshani et al., 2000; Shepherd et al., 2007). We have previously demonstrated that KCa3.1 has a key role in mediating scratch-induced migration of reactive astrocytes via the c-Jun/JNK MAPK signaling pathway (Yi et al., 2016a). Blockade of the KCa3.1 channel has also been shown to attenuate the Aβ-induced JNK MAPK pathway, resulting in downregulation of the inflammatory factors IL-1β, TNF-α, iNOS and COX-2 in the brain in a mouse model of Alzheimer’s disease (AD; Wei et al., 2016; Yi et al., 2016b). KCa3.1 plays an important role in the phenotypic switch of reactive astrocytes during the development of AD and blockade or gene deletion of KCa3.1 attenuated gliosis and neuron loss in a mouse model of AD (Wei et al., 2016). Microglia isolated from the infarcted area of mice subjected to middle cerebral artery occlusion (MCAO) exhibited higher densities of KCa3.1 currents than those from control mouse brains (Chen Y.-J. et al., 2016). Endothelial cells of the blood-brain barrier also exhibited KCa3.1 activity in rat brains following ischemic stroke and blockade of KCa3.1 attenuated cytotoxic edema by decreasing Na^+^ uptake (Chen et al., 2015).

The endoplasmic reticulum (ER) is the subcellular organelle that regulates intracellular calcium homeostasis and protein folding and an increasing number of studies have demonstrated that ER stress is responsible for ischemia-induced cellular dysfunction. Ischemia triggers the unfolded protein response (UPR) because of the disruption of protein folding in the ER (Yang and Paschen, 2016). Three ER pathways are activated in response to the UPR, including PKR-like ER kinase (PERK), activating transcription factor 6 (ATF6) and inositol-requiring enzyme 1 (IRE1; Gupta et al., 2016). During ER dysfunction, 78 kDa glucose-regulated protein (GRP78) dissociates from PERK, ATF6 and IRE1 and finally initiates proapoptotic signaling by activating CHOP. Pharmacological agents that maintain calcium homeostasis play an important role in regulating ER stress-induced apoptosis. It has been reported that blockade or genetic deletion of KCa3.1 protects against cisplatin-induced acute kidney injury by attenuation of ER stress and intrinsic apoptosis (Chen C.-L. et al., 2016).

Here, we present evidence that KCa3.1 represents a valid pharmacological target for modulation of phenotype during astrogliosis caused by ischemic stroke, in both oxygen–glucose deprivation (OGD)-treated astrocytes and in the brains of mice subjected to permanent middle cerebral artery occlusion (pMCAO). We found that both OGD and pMCAO upregulated KCa3.1, with a concomitant increase in the expression of glial fibrillary acidic protein (GFAP), a marker of reactive astrocytes. Gene deletion or pharmacological blockade of KCa3.1 was shown to suppress phenotypic modulation of astrocytes by inhibiting OGD-induced ER stress and JNK and ERK signaling pathways.

## Materials and Methods

### Animals

Male mice (10- to 12-week-old, 25–30 g) were housed in the Animal Research Center of Shanghai Jiao Tong University School of Medicine under a 12 h light/dark cycle with 23 ± 2°C and of 55 ± 5% (humidity). The experimental protocols were approved by the Animal Care and Use Committee of Shanghai Jiao Tong University School of Medicine, Shanghai, China (ethics protocol number: A-2015-010). All experiments were conducted in compliance with the ARRIVE guidelines. KCa3.1^−/−^ mice (Jackson Laboratory) mice have been described previously (Wei et al., 2016; Yi et al., 2016a). KCa3.1^−/−^ mice (KO) and their wildtype (WT) controls were on a C57BL/6J background.

### Permanent Focal Cerebral Ischemia

A pMCAO was induced under 2% chloral hydrate anesthesia as previously described (Chu et al., 2016). Body temperature was maintained at 37°C ± 0.5°C throughout the surgery using a thermostatically controlled heating pad and lamp (ALC-HTP, Shanghai Alcott Biotech Co., Ltd., Shanghai, China).

Briefly, a midline incision was made above the thyroid gland to expose the left common carotid artery (CCA). The external carotid artery (ECA) was ligated distal to the bifurcation of the CCA. A silicon-coated nylon suture, with a tip diameter of 0.22 mm (L2000, AAA, Guangzhou Jialing Biotech Co., Ltd., Guangzhou, China), was inserted into the left ECA. To monitor cerebral blood flow (CBF), a transcranial moor VMS-LDF2 laser Doppler blood flow and temperature monitor (Moor Instruments, Axminster, UK) was used. Until mice regained consciousness (about 1–2 h), a flexible fiber-optic probe of laser Doppler affixed to the skull was removed. The intraluminal suture was left *in situ* for different time points (1, 3, 6, 24 h). The filament was tied in place permanently. Mice were returned to the pathogen-free cages, and monitored till the end of the different time points (1, 3, 6, 24 h). Groups of mice were euthanized at 1, 3, 6 and 24 h after pMCAO. Brains were quickly collected for Western blotting or infarct volume analyses.

### Stroke Study Population and Quality Control

The operators were not involved in data analysis and acquisition. The observers performed the surgeries and parameters evaluation were unaware of the group to which each mouse belonged. The following conditions excluded mice from end-point analyses (exclusion criteria): (1) <80% reduction in CBF; (2) subarachnoid hemorrhage or the brain parenchyma bleeding (as macroscopically assessed during brain sampling); and (3) operation time >10 min. In total, 90 mice (45 C57BL/6 WT, 45 KO) were used in this study. Of the 90 mice subjected to pMCAO, nine mice (10%) met at least one exclusion criterion after randomization and, therefore, were withdrawn from the study.

### Determination of Infarct Volume

Groups of mice were sacrificed 3, 6 and 24 h after pMCAO, respectively. Brains were quickly removed and sectioned into five 1-mm-thick coronal slices starting from the frontal pole. All sections were stained with 2% triphenyltetrazolium chloride (TTC, Sigma-Aldrich) for 20 min at 37°C to visualize the infarctions (König et al., 2013). Finally 4% paraformaldehyde was used to fix the slices overnight at 4°C. ImageJ was used to measure the infarct area of each slice.

### Primary Culture of Astrocytes

Primary cerebral cortical astrocytes were obtained from 1 day to 2 day old neonatal WT or KO C57BL/6 mouse brains as previously described (Yu et al., 2014). Briefly, the cerebral cortices were removed and dissociated into single cell suspensions. Astrocytes were seeded into T75 flasks coated with poly-D-lysine hydrobromide (Sigma, St. Louis, MO, USA) in Dulbecco’s Modified Eagle’s Medium (DMEM, Sigma) containing 10% fetal bovine serum, 2 mM L-glutamine, 100 U/mL penicillin and 0.1 mg/ mL streptomycin at 37°C under a 5% CO_2_ atmosphere. When the astrocytes grew to confluence (10–14 days later), the cells were re-plated into six-well dishes in serum-containing medium. After reaching confluence, the cells were cultured in serum-free media for 24 h, and were then treated with OGD for different time points (1, 3, 4 6, 12 h). In some cases, astrocytes were pre-treated with TRAM-34 (Tocris Bioscience), the KCa3.1 channel blocker, 1 h before OGD.

### Oxygen–Glucose Deprivation

Confluent primary astrocytes were cultured in serum-free DMEM for 24 h before OGD, at which time the serum-free DMEM was replaced by glucose/glutamine-free DMEM. The serum-free and glucose/glutamine-free cultured astrocytes were first balanced for 30 min with 95% (v/v) N_2_, 5% (v/v) CO_2_ at 37°C before OGD. The cells were then placed in a hypoxia chamber filled with 95% (v/v) N_2_ and 5% (v/v) CO_2_ at 37°C for 1, 3, 4, 6, or 12 h.

### Western Blotting

Primary astrocytes or brain tissue extracts were lysed in a lysis buffer (50 mM Tris-HCl, 150 mM NaCl, 5 mM EDTA, 1% Triton X-100, 0.1% SDS, 1% sodium deoxycholate, 1 mM PMSF). The suspension was centrifuged at 12,000 rpm for 10 min at 4°C. Protein samples were separated by 12.5% sodium dodecyl sulfate-polyacrylamide gel electrophoresis (SDS-PAGE) and transferred to polyvinylidene fluoride (PVDF) membranes. The membranes were first incubated (overnight, 4°C) with the following primary antibodies: anti-total p38/JNK/ERK/c-Jun/eIF-2α, anti-phospho-p38/JNK/ERK/c-Jun/eIF-2α antibodies (1:1000, Cell Signaling Technology, Danvers, MA, USA), anti-GRP78 antibody (1:1000, Abcam), anti-GFAP antibody (1:5000, Dako, Glostrup, Denmark), anti-KCa3.1 antibody (1:500, Alomone Labs, Jerusalem, Israel) and anti-β-actin (1:1000, Sigma). The membranes were then incubated for 1 h at room temperature with appropriate horseradish peroxidase-conjugated secondary antibodies (1:3000; Amersham Biosciences). ImageJ software (National Institutes of Health) was used to quantify protein bands by densitometric analysis. β-Actin was used as an internal control for protein loading. All data were presented as fold increase normalized to the control group.

### Cell Viability

Cell viability was assessed using a Cell Counting Kit-8 (CCK-8, Dojindo Molecular Technologies) as previously described (Wei et al., 2016). Briefly, the astrocytes were plated into 96-well plate in serum-containing medium. After reaching confluence, the cells were cultured in serum-free media for 24 h, and were then treated with OGD for different time points (1, 3, 4 6, 12 h). Add 10 μl of CCK-8 to each well of the 96-well plate. Place in a CO_2_ incubator for 2 h to react. Measure the absorbance at 450 nm with a microplate reader.

### Statistical Analysis

All data are presented as means ± SEM. Statistical analyses were performed using Prism software (GraphPad Software, Inc., La Jolla, CA, USA). The Student’s *t*-test was used to compare differences between two groups and one-way analysis of variance (ANOVA) and Dunnett’s *post hoc* tests were used to compare differences from three or more groups. Statistical significance was set at *p* < 0.05.

## Results

### KCa3.1 Induction Is Associated with OGD-Induced Astrogliosis in Primary Astrocytes

**Figure 1:**
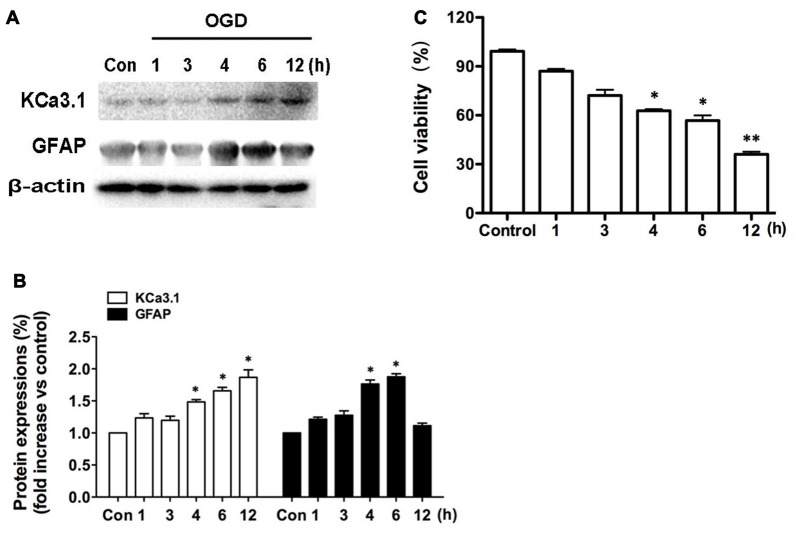
Up-regulation of KCa3.1 in OGD-induced reactive astrogliosis. Primary cultured astrocytes were subjected to OGD *in vitro* for different time periods, as shown. **(A)** Western blot analysis of astrocytic lysates following 1, 3, 4, 6 or 12 h of OGD, analyzed by antibodies to KCa3.1 and glial fibrillary acidic protein (GFAP). β-Actin was used as a loading control. **(B)** The *bar graphs* represent the fold changes of KCa3.1 and GFAP, normalized to control cells (*n* = 3). The data are expressed as means ± SEM. *p < 0.05 vs. the control cells. **(C)** Cell viability was determined using the CCK-8 assay. The values represent the percentage of cell viability induced by OGD (1, 3, 4, 6, 12 h) compared with the control group. *n* = 5. The data are expressed as means ± SEM. *p < 0.05, **p < 0.01 vs. the control cells. OGD, oxygen–glucose deprivation.

We recently reported (Wei et al., 2016) that KCa3.1 plays an important role in phenotypic switching of reactive astrocytes during the development of AD. Blockade or genetic deletion of KCa3.1 attenuated astrogliosis and loss of neurons in a mouse model of AD. The levels of KCa3.1 expression were determined over 12 h in OGD-induced astrogliosis *in vitro*. We also tested whether KCa3.1 was increased in the process of OGD-induced reactive astrogliosis *in vitro*. Western blots showed that OGD caused up-regulation of KCa3.1 in a time-dependent manner (1, 3, 4, 6, 12 h), as well as an increase in astrogliosis marker GFAP (Figures 1A,B). CCK-8 assay showed that OGD caused decrease in cell viability in a time-dependent manner (1, 3, 4, 6, 12 h; Figure 1C).

### KCa3.1 Is Involved in OGD-Induced Astrogliosis and ER Stress in Primary Astrocytes

**Figure 2:**
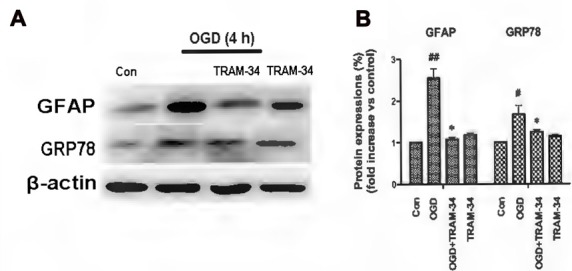
Blockade of KCa3.1 attenuated OGD-induced astrogliosis and endoplasmic reticulum (ER) stress in primary astrocytes. **(A)** Whole-cell lysates were collected to evaluate the expression of GFAP and 78 kDa glucose-regulated protein (GRP78) in primary astrocytes by western blotting after 4 h OGD. β-Actin was used as a loading control. **(B)** The *bar graphs* represent the ratio of GFAP/β-actin and GRP78/β-actin, normalized to the control (*n* = 3–4). β-Actin was used as a loading control. The data are expressed as means ± SEM. ^#^*p* < 0.05, ^##^*p* < 0.01 vs. control; *p < 0.05 vs. OGD. Con, control; OGD, oxygen–glucose deprivation.

The ER is a subcellular organelle that regulates intracellular calcium homeostasis and protein folding and it has been demonstrated that ER stress is responsible for ischemia-induced cell dysfunction (Verkhratsky and Petersen, 2002). Ischemia triggers the UPR because of the disruption of protein folding in the ER. It has been reported that blockade or genetic deletion of KCa3.1 protects against cisplatin-induced acute kidney injury by attenuating ER stress (Chen C.-L. et al., 2016). In this study, we tested whether KCa3.1 channels are involved in OGD-induced ER stress in astrogliosis. We found that OGD (4 h) upregulated expression of GFAP (*p* < 0.01; Figures 2A,B) and ER stress marker GRP78 (*p* < 0.05; Figures 2A,B), compared with control astrocytes. Up-regulation of GFAP was reduced by the KCa3.1 blocker TRAM-34 at a concentration of 1 μM (*p* < 0.05; Figures 2A,B) and TRAM-34 also significantly suppressed the effect of OGD on GRP78 production (*p* < 0.05; Figures 2A,B). Blockade of KCa3.1 thus reduced OGD-induced expression of GFAP and GRP78 in reactive astrocytes.

### Knockout of KCa3.1 Inhibited OGD-Induced ER Stress in Reactive Astrogliosis

**Figure 3:**
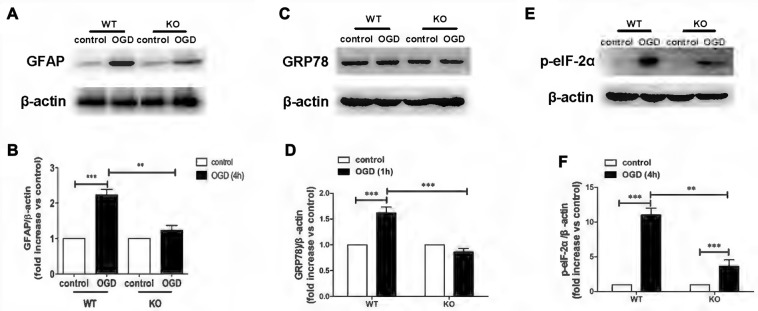
Genetic deletion of KCa3.1 attenuated OGD-induced ER stress in primary astrocytes. **(A,B)** Whole-cell lysates were collected to evaluate the expression of GFAP in wildtype (WT) and KCa3.1 KO astrocytes by western blotting after 4 h OGD. β-Actin was used as a loading control. The *bar graphs* represent the ratio of GFAP/β-actin, normalized to the control (*n* = 3–4). **(C,D)** Whole-cell lysates were collected to evaluate the expression of GRP78 in WT and KCa3.1 KO astrocytes by western blotting after 1 h OGD. β-Actin was used as a loading control. The *bar graphs* represent the ratio of GRP78/β-actin, normalized to the control (*n* = 3–4). **(E,F)** Whole-cell lysates were collected to evaluate the expression of p-eIF-2α in WT and KCa3.1 KO astrocytes by western blotting after 4 h OGD. β-Actin was used as a loading control. The *bar graphs* represent the ratio of p-eIF-2α/β-actin, normalized to the control (*n* = 3–4). The data are expressed as means ± SEM. **p < 0.01, ***p < 0.001. WT, wild type; KO, knockout; OGD, oxygen–glucose deprivation.

KO astrocytes were used to test whether KCa3.1 channels are indeed involved in OGD-induced ER stress in astrogliosis. Similarly to astrocytes treated with TRAM-34, KO astrocytes exhibited a decrease in GFAP (*p* < 0.01; Figures 3A,B), GRP78 (*p* < 0.001; Figures 3C,D) and phosphorylated eIF-2α protein expression (*p* < 0.01; Figures 3E,F), compared with WT astrocytes. Taken together, these data indicate that KCa3.1 might serve as a target to regulate ER stress in OGD-induced astrogliosis *in vitro*.

**Figure 4:**
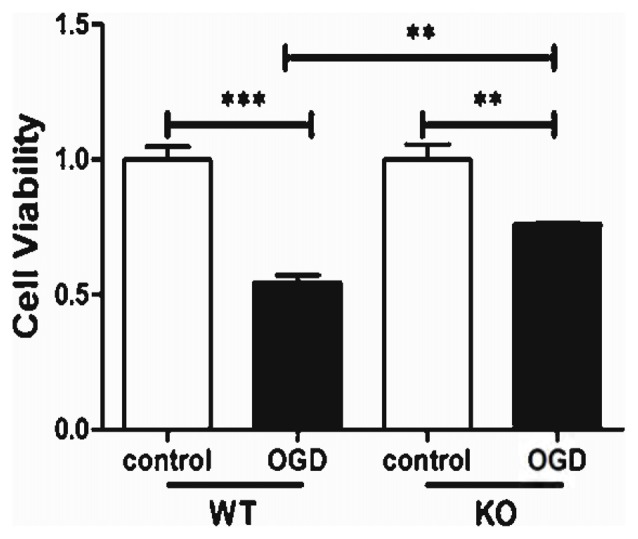
KCa3.1 channel is involved in OGD-induced reduction in viability of astrocytes. Cell viability was determined using the CCK-8 assay. The values represent the fold changes of cell viability induced by OGD. Genetic deletion of the KCa3.1 channel attenuated the decrease in viability of astrocytes after 4 h OGD, compared with WT cells (*n* = 5). The data are expressed as means ± SEM. **p < 0.01, ***p < 0.001. WT, wild type; KO, knockout; OGD, oxygen–glucose deprivation.

KO astrocytes were also used to evaluate whether KCa3.1 is involved in OGD-induced changes in astrocyte viability. Both WT and KO astrocytes were stimulated with or without 4 h OGD. The OGD-induced decrease in cell viability (CCK-8 assay) was attenuated in KO astrocytes compared with WT astrocytes (*p* < 0.01; Figure 4).

### Involvement of KCa3.1 in OGD-Induced ER Stress and Astrogliosis through the JNK and ERK1/2 Signaling Pathways

**Figure 5:**
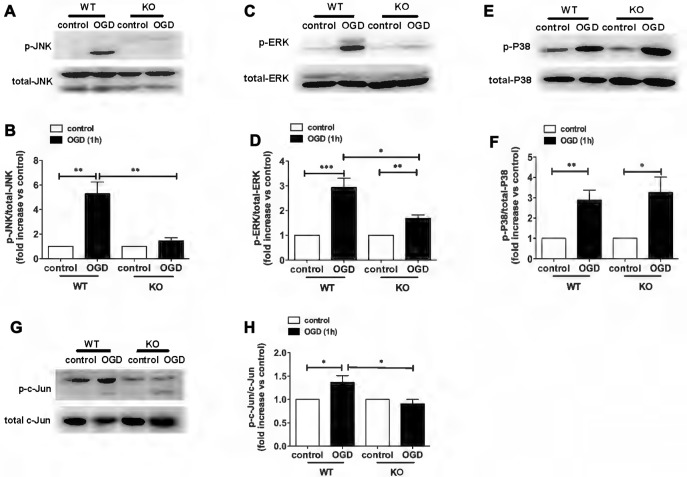
Involvement of KCa3.1 in OGD-induced phenotypic modulation of astrocytes through the ERK1/2 and JNK signaling pathways.** (A,C,E)** Representative images of total JNK, ERK1/2, P38 and phosphorylated JNK (p-JNK), ERK1/2 (p-ERK1/2), P38 (p-P38) in WT and KO astrocytes with or without OGD (1 h). **(B,D,F)** Mean values of p-JNK, p-ERK1/2 and p-P38 relative to total JNK, ERK1/2 and P38 protein (*n* = 3). Data are presented as means ± SEM. *p < 0.05, **p < 0.01, ***p < 0.001. **(G)** Representative images of total c-Jun and phosphorylated c-Jun (p-c-Jun) in WT and KO astrocytes, with or without OGD (1 h). **(H)** Mean values of p-c-Jun relative to total c-Jun protein (*n* = 3). Data are presented as means ± SEM. *p < 0.05. WT, wild type; KO, knockout; OGD, oxygen–glucose deprivation.

In a recent study, we demonstrated a key role for KCa3.1 in mediating scratch-induced migration of reactive astrocytes via the c-Jun/JNK MAPK signaling pathway (Yi et al., 2016a). We additionally showed that blockade of the KCa3.1 channel attenuated the Aβ-induced JNK MAPK pathway, resulting in down-regulation of inflammatory factors in the brain in a mouse model of AD (Wei et al., 2016). To understand the mechanisms by which pharmacological blockade or genetic deletion of KCa3.1 inhibits OGD-induced reactive astrogliosis and ER stress, we examined the effects of KCa3.1 on the MAPK signaling pathway, JNK, ERK and P38. The phosphorylation of JNK, ERK and P38 was studied by western blot (Figure 5). WT astrocytes exposed to OGD for 1 h showed a significant increase in phosphorylation of JNK (p-JNK; *p* < 0.01; Figures 5A,B), ERK1/2 (p-ERK1/2; *p* < 0.001; Figures 5C,D) and P38 (p-P38; *p* < 0.01; Figures 5E,F). Compared with WT cells, phosphorylation was attenuated in OGD-stimulated KO astrocytes (Figures 5A–D), except for p-P38 (Figures 5E,F). As the downstream pathway of MAPK/JNK, phosphorylation of c-Jun was also tested in both WT and KO astrocytes, with or without OGD stimulation (Figures 5G,H). KO astrocytes exhibited reduced phosphorylation of c-Jun (*p* < 0.05; Figures 5G,H) in response to OGD compared with astrocytes from WT mice.

### Genetic KCa3.1 Deletion Reduces Infarct Area in pMCAO

Recently it was reported that in a mouse model of ischemia, wild type mice treated with the KCa3.1 blocker TRAM-34 resulted in a decrease in infarct areas (Chen Y.-J. et al., 2016).

**Figure 6:**
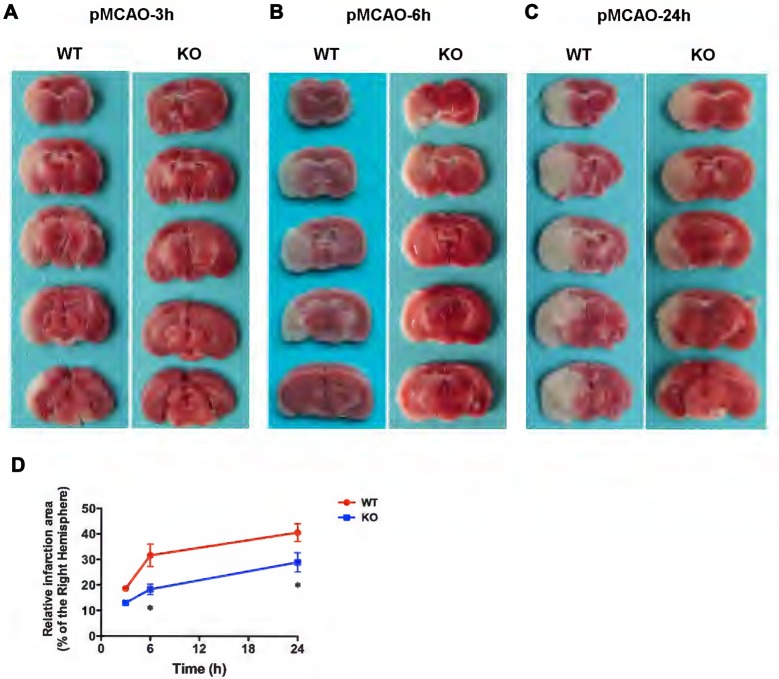
KCa3.1 deficiency reduces infarction volume. Focal cerebral ischemia was induced by permanent middle cerebral artery occlusion (pMCAO). **(A–C)** Representative TTC staining of five corresponding coronal brain sections of WT and KO mouse after 3 h **(A)**, 6 h **(B)** and 24 h **(C)** of pMCAO. **(D)** Quantitative analysis of infarction volume in 3, 6 and 24 h, respectively. Data are presented as means ± SEM. *n* = 5. *p < 0.05, compared with ischemic WT group.

In the present study, WT and KO mice were subjected to 3, 6 and 24 h of pMCAO and infarct volumes were assessed (Figure 6). As shown in Figures 6A–D, TTC staining of pMCAO brains indicated that infarct volumes in KO mice was smaller than those in WT mice, and infarct areas in KO mice were significantly reduced at 6 h (Figure 6B) and 24 h (Figure 6C) after pMCAO compared with those in WT mice (*p* < 0.05, Figure 6D) but there was no significant difference in infarct measurement at 3 h (*p* > 0.05, Figures 6A,D).

### KCa3.1 Deficiency Attenuates ER Stress and Astrogliosis through JNK and ERK1/2 Signaling Pathways in *KCa3.1*^−/−^ pMCAO Mice

**Figure 7:**
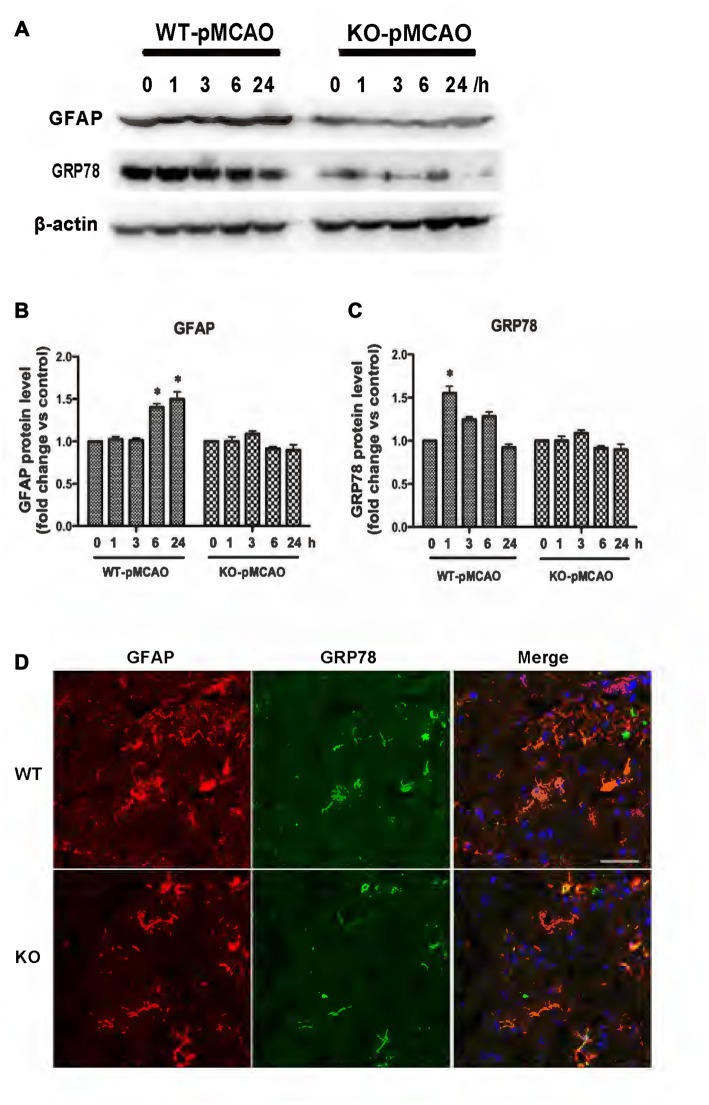
KCa3.1 is involved in ER stress *in vivo*. Focal cerebral ischemia was induced by pMCAO in WT and KO mice. **(A–C)** Western blot analysis of lysates from 10-week-old male WT and KO mice 1, 3, 6 and 24 h after pMCAO, analyzed by antibodies to GFAP and GRP78. Data represent means ± SEM of GFAP and GRP78 density, normalized to β-actin values (*n* = 3). *p < 0.05 compared with control, 0 h. **(D)** Double immunofluorescence staining of GRP78 (green) with GFAP (red) in brain sections of WT and KO pMCAO (6 h) mice. Nuclei were stained in blue with DAPI. Scale bar: 25 μm. WT, wild type; KO, knockout.

KCa3.1 levels in the brain have been reported to increase during astrogliosis in a mouse model of ischemic stroke and genetic deletion of KCa3.1 reduced gliosis and neuronal loss in this mouse model (Wei et al., 2016). Here, a pMCAO mouse model was used as previously described (Chu et al., 2016). To determine whether OGD-induced ER stress and the phenotypic switching that accompanies astrogliosis in primary cultured astrocytes also occur *in vivo*, we investigated expression levels of GFAP and GRP78 proteins in the brains of both WT and KO pMCAO mice. Western blots showed that expression levels of both GFAP (Figures 7A,B) and GRP78 (Figures 7A,C) proteins were significantly elevated in the brains of WT pMCAO mice following MCAO blockage, whereas expression levels of these proteins were significantly lower in the brains of KO pMCAO mice (Figures 7A–C). Co-immunostaining of GRP78 with astrocytes specific marker GFAP was performed on brain sections of WT and KO pMCAO mice. In KO pMCAO mice brain, lower expression of GRP78 was detected in GFAP^+^ astrocytes than that in WT group mice (Figure 7D). Overall, these *in vivo* results are consistent with those reported for primary cultured astrocytes *in vitro*, demonstrating that ischemic stroke initiated ER stress and astrogliosis in both the OGD-induced astrocytes and the brains in the pMCAO mouse model.

**Figure 8:**
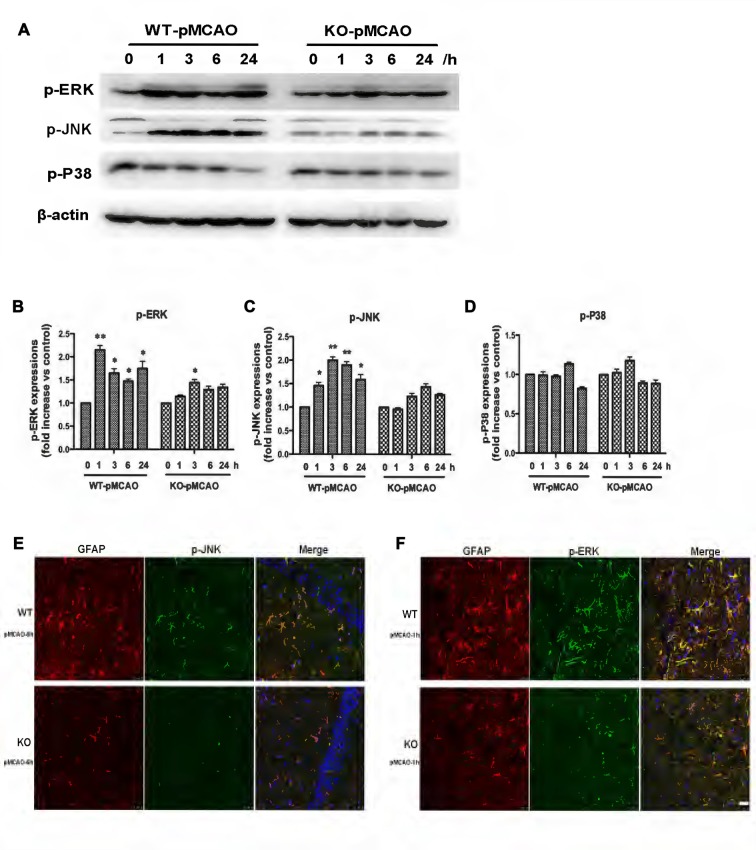
Involvement of KCa3.1 in pMCAO mouse model through ERK1/2 and JNK signaling pathways. Focal cerebral ischemia was induced by pMCAO in WT and KO mice. **(A–D)** Western blot analysis of lysates from 10-week-old male WT and KO mice 1, 3, 6 and 24 h after pMCAO, analyzed by antibodies to phosphorylated ERK1/2 (p-ERK1/2), JNK (p-JNK), P38 (p-P38). Data represent means ± SEM of p-ERK1/2, p-JNK, p-P38 density, normalized to β-actin values (*n* = 3). *p < 0.05, **p < 0.01 compared with control, 0 h. **(E,F)** Double immunofluorescence staining of GFAP (red) with p-JNK (green) and p-ERK1/2 (green) in brain sections of of WT and KO pMCAO mice. **(E)** Co-staining of GFAP with p-JNK in brain sections of WT and KO pMCAO (6 h) mice; **(F)** Co-staining of GFAP with p-ERK in brain sections of WT and KO pMCAO (1 h) mice. Nuclei were stained in blue with DAPI. Scale bar: 25 μm. WT, wild type; KO, knockout.

OGD induced upregulation of the KCa3.1 channel and astrogliosis via activation of the JNK and ERK1/2 signaling cascades *in vitro*, as described above. To provide direct evidence that KCa3.1 channels participated in ischemic stroke via the ERK1/2 and JNK signaling pathways *in vivo*, we examined the activation of the ERK1/2 (Figures 8A,B) and JNK (Figures 8A,C) signaling pathways following MCAO blockage for up to 24 h in WT and KO mice. Both p-JNK and p-ERK1/2 levels, but not p-P38 levels (Figures 8A,D), were significantly higher in WT pMCAO mice than in KO pMCAO mice. Co-immunostaining of p-JNK or p-ERK with astrocytes specific marker GFAP was performed on brain sections of WT and KO pMCAO mice. In KO pMCAO mice brain, lower expression of p-JNK (Figure 8E) or p-ERK (Figure 8F) was detected in GFAP^+^ astrocytes than that in WT group mice (Figures 8E,F). The expressions of MAPK signal pathways and GFAP from sham WT and KO mice were shown in Supplementary Figure S1.

## Discussion

The major findings of the present study are that both genetic deletion *in vivo* and pharmacological blockade of KCa3.1 *in vitro* inhibit upregulation of astrogliosis marker GFAP, which is accompanied by decreased ER stress markers GRP78 and eIF-2α, in both OGD-induced astrocytes and in the pMCAO mouse model of ischemic stroke. Additionally, OGD-induced upregulation of KCa3.1 and astrogliosis were regulated by the JNK and ERK signaling pathways. These findings demonstrate a novel role for the KCa3.1 channel in regulating phenotypic modulation of astrocytes through the process of ER stress and the MAPK/JNK and ERK signaling pathways.

CNS insults, such as ischemia, trauma and neurodegenerative disease, trigger neurotoxicity and gliosis that can rapidly cause reactive astrogliosis (Sofroniew, 2005; Jha et al., 2014). The upregulation of GFAP during astrogliosis after stroke is a spontaneous response to CNS insults. In our study, western blot analysis showed that upregulation of GFAP and KCa3.1 was concomitant with increases in ER stress markers GRP78 and eIF-2α after pMCAO.

It has been reported that KCa3.1 is involved in the activation of microglia and that activated microglia induce neurotoxicity *in vitro* (Kaushal et al., 2007; Ferreira et al., 2014). Higher current densities and stronger KCa3.1 immunoreactivity were detected in activated microglia from the brains of pMCAO mice than from the brains of control animals (Chen Y.-J. et al., 2016). We have previously demonstrated that genetic deletion or pharmacological blockade of KCa3.1 channels reduced gliosis and loss of neurons in a mouse model of AD (Wei et al., 2016; Yi et al., 2016b). Levels of ER stress markersGRP78, eIF-2α and astrogliosis marker GFAP were all significantly lower in OGD-stimulated KO astrocytes than in WT cells, suggesting that downregulation of these markers might indicate reduced astrogliosis and thus less astrogliosis-induced neuronal damage.

Ischemic stroke, which involves a cascade of events such as inflammation, calcium overload and oxidative stress leading to cell apoptosis (Mehta et al., 2007; Barone et al., 2009), is the second most common cause of death and disability worldwide. The JNK/c-Jun signaling pathway has been reported to regulate inflammatory reactions in the brain. JNKs, known as stress-activated protein kinases, are a sub-family of MAP kinases whose activation is associated with different stresses, such as stimulation by cytokines and oxidative stress (Bendinelli et al., 1996; Li et al., 2002). Activated JNK phosphorylates downstream substrate c-Jun, which induces the apoptotic signaling pathway (Anand and Babu, 2011). JNK/c-Jun plays an important role during stress-induced cell death in the brain during ischemic stroke and blockade of this pathway may thus be neuroprotective (Borsello et al., 2003; Carboni et al., 2004; Gao et al., 2005). In a recent study, we demonstrated that blockade of KCa3.1 inhibited Aβ-induced indirect neurotoxicity via activation of JNK during astrogliosis (Wei et al., 2016). Upregulation of proinflammatory factors and cytokines, such as IL-1β, ROS and TNF-α, were inhibited by TRAM-34 in the APP/PS1 mouse model of AD. We also demonstrated that both blockade and genetic deletion of KCa3.1 reduced the sharp rise in astrocytic [Ca^2+^]_i_ and attenuated the phosphorylation of JNK and c-Jun proteins that were induced by scratch injuries (Yi et al., 2016a).

It has been reported that activation of JNK signaling pathways contributes to ischemia-induced oxidative stress in ischemic kidney cell death (Yang et al., 2016). The UPR has been observed in the ischemia brain. Reduced [K^+^]_i_ is associated with the activation of caspases and apoptosis (Kondratskyi et al., 2015). Increased [Ca^2+^]_i_ activates KCa3.1 and results in K^+^ efflux, which is involved in apoptosis of lymphocytes and erythrocytes. Recently, Chen C.-L. et al. (2016) reported that blockade of KCa3.1 protected against cisplatin-induced acute kidney injury through the attenuation of apoptosis by interference with intrinsic apoptotic and ER stress-related mediators.

In summary, the present study demonstrates that, in OGD-induced astrogliosis and pMCAO mouse brain: (1) ER stress compromises the role of KCa3.1 channels in astrogliosis; (2) OGD induces astrogliosis through MAPK/JNK/c-Jun and ERK signaling pathways involving ER stress-mediated inhibition of KCa3.1 channels.

## Author Contributions

ZY supervised the entire project, designed research and wrote the article. HC conceived and designed the experiments, interpreted and analyzed data, supervised all the experimental procedure. MY and TW conceived and designed the experiments, performed research, interpreted and analyzed data. XG analyzed data.

## Conflict of Interest Statement

The authors declare that the research was conducted in the absence of any commercial or financial relationships that could be construed as a potential conflict of interest.

## Supplementary Material

FIGURE S1 The expressions of MAPK signal pathways and glial fibrillary acidic protein (GFAP) from sham WT and KO mice. **(A–D)** Western blot analysis of lysates from sham WT and KO mice, analyzed by antibodies to **(A)** phosphorylated JNK (p-JNK), **(B)** phosphorylated ERK1/2 (p-ERK1/2), **(C)** phosphorylated P38 (p-P38) and **(D)** GFAP. Data represent means ± SEM of p-JNK, p-ERK1/2, p-P38 and GFAP density, normalized to total JNK, total ERK1/2, total P38 and β-actin values (*n* = 3). WT, wild type; KO, knockout.

## Abbreviations

ATF6: activating transcription factor 6
ER: endoplasmic reticulum
GFAP: glial fibrillary acidic protein
GRP78: 78 kDa glucose-regulated protein
IRE1: inositol requiring enzyme 1
KCa3.1: intermediate-conductance calcium-activated potassium channel
PERK: PKR-like endoplasmic reticulum kinase
pMCAO: permanent middle cerebral artery occlusion
tPA: tissue plasminogen activator
TRAM-34: 1-((2-chlorophenyl) (diphenyl) methyl)-1H-pyrazole
UPR: unfolded protein response.
