# Supplementary figures and images for: KCa3.1 Inhibition Switches the Astrocyte Phenotype during Astrogliosis Associated with Ischemic Stroke Via Endoplasmic Reticulum Stress and MAPK Signaling Pathways
Source: Front Cell Neurosci. 2017 Oct 12;11:319. doi: 10.3389/fncel.2017.00319 (PMC5643415; doi:10.3389/fncel.2017.00319)

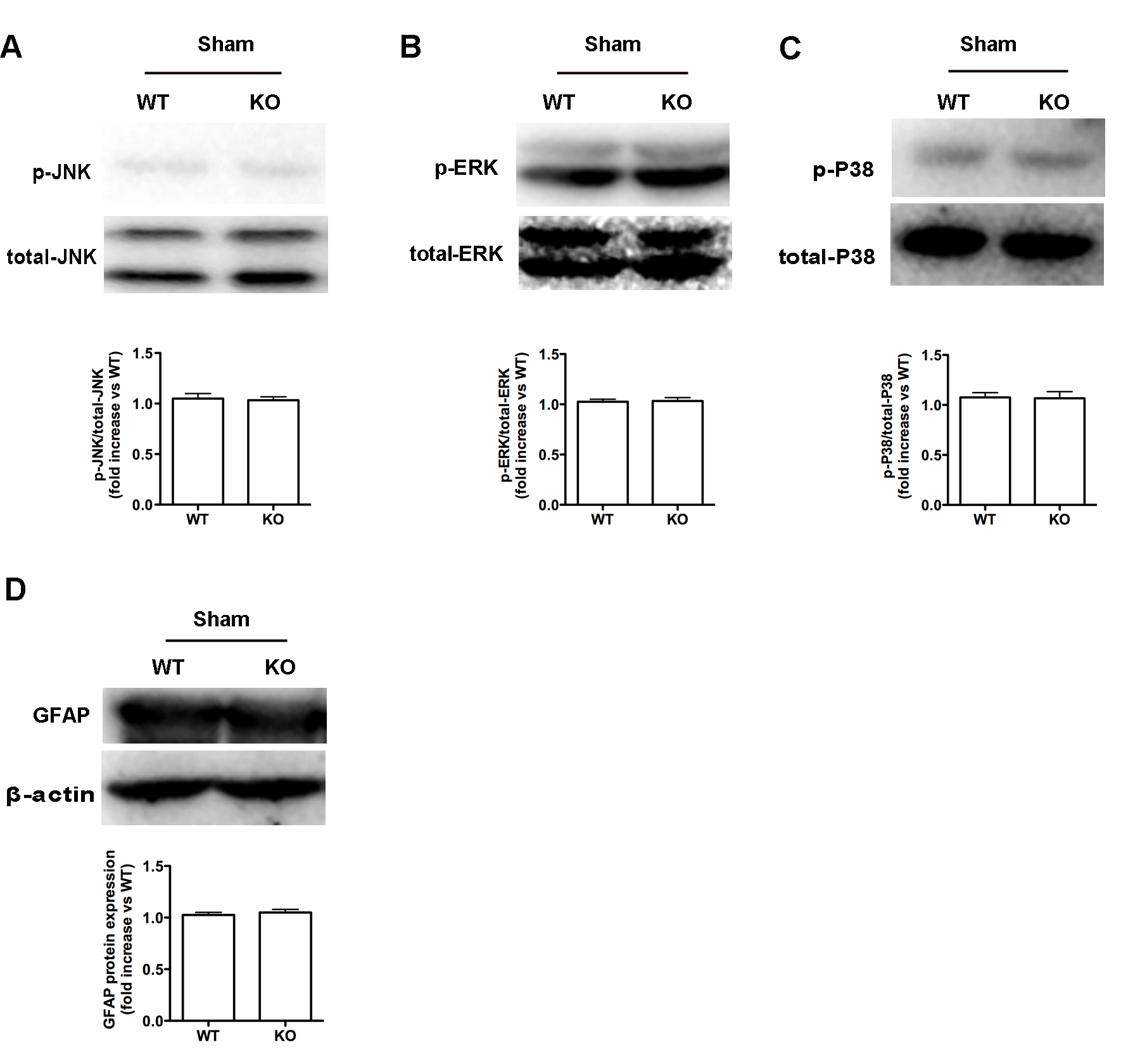

Supplement: FIGURE S1 — The expressions of MAPK signal pathways and glial fibrillary acidic protein (GFAP) from sham WT and KO mice. (A–D) Western blot analysis of lysates from sham WT and KO mice, analyzed by antibodies to (A) phosphorylated JNK (p-JNK), (B) phosphorylated ERK1/2 (p-ERK1/2), (C) phosphorylated P38 (p-P38) and (D) GFAP. Data represent means ± SEM of p-JNK, p-ERK1/2, p-P38 and GFAP density, normalized to total JNK, total ERK1/2, total P38 and β-actin values (n = 3). WT, wild type; KO, knockout. [file Image_1.tif]
